# Advances in Fracture Fixation: Enhancing Stability and Promoting Healing

**DOI:** 10.7759/cureus.88595

**Published:** 2025-07-23

**Authors:** Muhammad Rizwan Umer, Areeba Asghar, Muhammad Abu Zar Nawaz, Fokehah Maryam, Safeer Ahmad Javid, Muddasir Reyaz Hassan, Amna Akbar, Areeba Zahid, Mariam K

**Affiliations:** 1 Trauma Surgery, Royal Sussex County Hospital, Brighton, GBR; 2 Medicine and Surgery, Russells Hall Hospital, Dudley Group NHS Foundation Trust, Dudley, GBR; 3 Medicine and Surgery, Royal Devon Healthcare NHS Foundation Trust, England, GBR; 4 Medicine and Surgery, Tehsil Headquarters Hospital, Bhalwal, PAK; 5 Medicine and Surgery, Northwick Park Hospital, London, GBR; 6 Emergency and Accident, Abbas Institute of Medical Sciences, Muzaffarabad, PAK; 7 Medicine and Surgery, Faisalabad Medical University, Faisalabad, PAK; 8 Medicine and Surgery, Hamdard University, Karachi, PAK

**Keywords:** bioresorbable implants, bone healing, fracture fixation, intramedullary nails, locking plates, orthopedic surgery, osteoporosis

## Abstract

Advancements in fracture fixation have significantly reshaped orthopedic trauma management by improving mechanical stability and promoting biological healing. This retrospective observational study assessed clinical and biomechanical outcomes in 500 patients who underwent surgical fixation for radiologically confirmed fractures. Patients were categorized into two groups: Group A received conventional fixation methods (e.g., standard plates and screws), while Group B was treated with advanced systems, including locking plates, intramedullary nails, minimally invasive percutaneous osteosynthesis (MIPO), and bioresorbable implants. Data analysis included 50 variables spanning demographics, clinical profiles, imaging, laboratory results, implant details, and postoperative outcomes. Statistical methods such as t-tests, chi-square, Mann-Whitney U, Kruskal-Wallis, Kaplan-Meier survival analysis, and multiple regression models were performed using IBM SPSS Statistics for Windows, Version 27.0 (Released 2019; IBM Corp., Armonk, NY, US). The study found that advanced fixation techniques resulted in significantly faster healing (12.4 vs. 14.9 weeks, p < 0.001), earlier mobilization (p = 0.003), and better three-month functional outcomes, with 80.1% in Group B achieving good to excellent recovery compared to 58.2% in Group A (p < 0.001). The complication rate was notably lower in the advanced group (10.8% vs. 20.4%, p = 0.003). Regression analysis identified implant type, vitamin D levels, and diabetes status as key predictors of healing duration, complication risk, and functional recovery. Predictive models demonstrated strong performance: a linear regression model for healing time (R² = 0.38, p < 0.001), a logistic regression model for complications (accuracy = 71.6%), and an ordinal regression model for functional outcomes (Nagelkerke R² = 0.41, p < 0.001). These findings support the clinical benefits of modern fixation systems and highlight the importance of integrating biomechanical choices with patient-specific health indicators to enhance fracture management outcomes.

## Introduction

Fracture fixation remains a critical pillar of orthopedic trauma care, essential for restoring bone continuity, ensuring mechanical stability, and promoting healing [1]. Globally, fractures account for a significant portion of the injury burden, with over 178 million new fracture cases reported annually [2]. This number is expected to grow due to increasing life expectancy, urbanization, and the rising incidence of road traffic accidents. In fact, road traffic accidents are responsible for approximately 20-50 million injuries worldwide each year, with traumatic fractures being among the most common outcomes. According to the World Health Organization (WHO), road injuries rank as one of the top 10 causes of disability-adjusted life years (DALYs), contributing significantly to global morbidity and mortality. Simultaneously, the aging global population has led to a surge in osteoporotic fractures, especially hip, spine, and wrist fractures [3]. Currently, more than 200 million people worldwide suffer from osteoporosis, a condition characterized by weakened bones and an increased risk of fractures [2]. One in three women and one in five men over the age of 50 are likely to experience an osteoporotic fracture in their lifetime due to the prevalence of bone fragility associated with the condition. This dual burden of traumatic and fragility-related fractures demands innovative and adaptable fixation strategies to improve healing outcomes across age groups and demographics [2,4].

Fracture pathology is complex and multifactorial. In the younger adult population, fractures are most often produced from high-energy trauma in the form of falls, sports injuries, or motor vehicle accidents, resulting in complex fractures with soft tissue involvement that usually occur in multiple bones [5]. Conversely, the elderly population experiences low-energy fractures, secondary to bone fragility, which take the form of fragility fractures. These, in turn, may also create a fragmented pattern of fractures. Osteoporotic bone can become brittle due to decreased bone mineral density, leading to fractures from low-energy mechanisms, such as falls from standing height [6]. Pathological fractures caused by cancer or metabolic bone disease can occur in both young and elderly populations, but the pathology of the bone complicates the fracture. The aforementioned mechanisms of a fracture demonstrate the need for fracture fixation systems that are mechanically stable and, at the same time, provide biological support [7].

Historically, fractures were treated primarily with non-operative care, including casting and skeletal traction. Although sometimes effective, both resulted in extended immobilization, with either malunion or a delayed return to function [8]. The introduction of modern internal fixation marked a turning point, as patients began to demonstrate good functional outcomes. Internal fixation, achieved through the use of plates, screws, intramedullary nails, and external fixators, introduced a new method for treating fractures that provides precise alignment and stabilization, allowing for early mobilization [9]. In a relatively short period, the state of the art has advanced significantly further. Locking compression plates (LCPs), for example, provide angular stability and are very useful in osteoporotic or comminuted fractures. Intramedullary nails, especially in the femur and tibia, equal or exceed load sharing and decrease healing time [10].

The benefits of minimally invasive surgical techniques have expanded. They are gentler to the surrounding soft tissue and blood supply, thus decreasing the risk of infection and providing a quicker recovery. The development of fracture fixation techniques that employ 3D printing, custom implants, and bioresorbable materials has revolutionized the field of fracture care, leading to a more patient-specific approach to treatment [11]. The use of biological augmentation is another emerging area of consideration for fracture healing. In the area of healing biology, bone grafts, stem cells, and growth factors have formed the biological environment for healing. Despite these advances, national databases on fractures suggest that 5%-10% of fractures treated surgically develop a complication, such as non-union or implant failure, especially in higher-risk patients [12,13].

This brings us to the issues that are addressed in this study. Technology has advanced rapidly, but we are still unable to achieve optimal healing in all types of fractures and for patients with various underlying conditions. The fixation of osteoporotic bone, management of complex fractures, and treatment of open injuries have all demonstrated that current practices have limitations [14]. The issue of cost and the inability to access advanced surgical techniques will exacerbate these problems, especially in low- and middle-income countries, which is a real-world problem of unequal access to fracture care [15].

The primary purpose of this undertaking is to research recent developments in methods of fixation that combine mechanical stability and biological healing. The specific purposes of this study are to assess the historical progression of the design and clinical performance of contemporary fixation systems, survey the invigoration of new technologies such as locking plates and bioabsorbable materials, incorporate and detail fixation complications associated with traditional and modern fixation, and provide evidence-based practical recommendations, as best as we can, for fixation according to fracture type and quality of bone as well as healthcare environment. A collaborative integration of biomechanical, clinical, and technological aspects of fixation will develop a more effective, unequivocal, and patient-centric approach to fracture management.

## Materials and methods

Study design

A retrospective observational study evaluated the clinical and biomechanical outcomes following fracture fixation using various fixation methods, including modern techniques such as locking plates, intramedullary nails, and bioresorbable implants. This study aims to evaluate the clinical effectiveness of these advanced systems compared to traditional fixation techniques, which provided maximum stability while allowing for as much biological healing as possible, along with minimal complications. Data were collected from the electronic health records of the orthopedic units in the study hospitals over a descriptive period.

Study population

The study consisted of 500 patients who underwent surgical fixation for fractures of various types, including 200 femur fractures, 150 tibia fractures, 100 humerus fractures, and 50 forearm fractures. This breakdown highlights the sample's heterogeneity, encompassing a diverse range of fracture locations and mechanisms of injury, which is crucial for assessing the applicability of the results to different patient populations. The participants included a variety of demographics and clinical groups, including adult trauma patients, elderly persons, osteoporotic cases, and polytrauma. This broad inclusion will allow the results to be applicable across clinical environments and fracture types.

Inclusion and exclusion criteria

Patients were eligible if they were 18 years or older, had radiologically confirmed fractures, and had undergone surgical intervention using internal or external fixation techniques. Additional criteria included a minimum of three months of follow-up and complete clinical and outcome documentation. Exclusion criteria were set to eliminate pediatric cases, patients managed non-operatively, and those with incomplete records or follow-up data below the threshold duration.

Data collection

A structured data collection sheet was used to capture 50 variables covering demographic data (age, sex, BMI, lifestyle), clinical information (fracture type, location, mechanism, ASA score), diagnostic results (X-ray, CT, DEXA), laboratory values (hemoglobin, WBC count, CRP, vitamin D, serum calcium), treatment history (previous fractures, surgical approach, duration), implant characteristics (type, screw configuration, plate length), and comorbid conditions (diabetes, cardiovascular disease, osteoporosis). Outcomes of interest included fracture union time, incidence of complications, time to weight bearing, reoperation, length of hospital stay, and functional recovery measured on an ordinal scale.

Grouping and interventions

Patients were divided into two groups based on the type of fixation received. Group A included those treated with conventional fixation methods such as standard plates and screws. Group B comprised patients managed using advanced fixation techniques, including locking compression plates, intramedullary nails, minimally invasive percutaneous osteosynthesis (MIPO), and bioresorbable implants. Inter-group comparisons were made across healing time, complication rates, reoperation frequency, and functional outcomes.

Exploratory data analysis (EDA)

Initial exploratory data analysis was conducted to understand variable distributions, detect outliers, and determine the appropriate statistical tests. Histograms, boxplots, and scatterplots were used for visualization, while Shapiro-Wilk tests evaluated the normality of continuous variables. Cross-tabulations helped identify preliminary trends between categorical variables such as implant type and complication rates. Missing data analysis and heatmaps were also used to examine data integrity and inter-variable relationships.

Statistical analysis

All statistical analyses were performed using IBM SPSS Statistics for Windows, Version 27.0 (Released 2019; IBM Corp., Armonk, NY, US). Descriptive statistics, including means, medians, and standard deviations, were calculated for continuous variables, while frequencies and percentages were used for categorical data. Inferential tests included independent samples t-tests and Mann-Whitney U tests for two-group comparisons, and ANOVA or Kruskal-Wallis tests for comparisons across multiple groups. Chi-square tests assessed associations between categorical variables. Regression models were employed to identify predictors of healing and complications, including binary logistic regression for dichotomous outcomes, linear regression for continuous outcomes such as healing time, and ordinal logistic regression for ranked outcomes like functional status. The model-building process involved stepwise selection to identify the most significant predictors, with covariates chosen based on clinical relevance and statistical significance. To assess the robustness of the models, internal validation was performed using k-fold cross-validation, ensuring the stability and generalizability of the findings. Survival analyses using Kaplan-Meier curves and Cox regression were performed to evaluate time-to-union and other event-based outcomes. Statistical significance was set at p < 0.05.

Software tools

Data preprocessing and preliminary analysis were conducted in Microsoft Excel (Microsoft Corp., Redmond, WA, US). IBM SPSS Statistics for Windows, Version 27.0, was used for statistical testing, while Python 3.0 (with libraries like Pandas, Matplotlib, and Seaborn) (Python Software Foundation, Fredericksburg, VA, US) was used for exploratory analytics and data visualization.

Ethical considerations

All patient data were anonymized, and institutional review board approval was obtained before data analysis. The study complied with the ethical principles of the Declaration of Helsinki and maintained strict confidentiality under data protection guidelines.

## Results

Demographic and baseline characteristics

**Figure 1:**
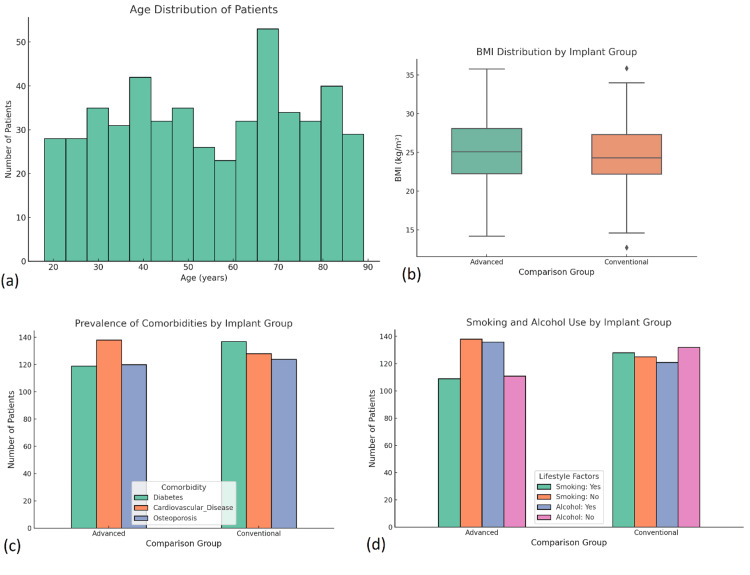
Demographic and lifestyle characteristics of the study population. (a) Histogram showing the age distribution of patients undergoing surgical fracture fixation, indicating a broad age range, with a peak around 65-70 years. (b) Box plot comparing BMI distributions between patients in the advanced and conventional implant groups. Median BMI values were similar across groups, with comparable interquartile ranges. Statistical comparison was performed using an independent samples t-test (p = 0.42), with no significant difference observed. (c) Bar chart displaying the prevalence of major comorbidities (diabetes, cardiovascular disease, and osteoporosis) by implant group. Differences were assessed using the chi-square test. Osteoporosis prevalence showed a significant difference (p = 0.04), while others were not statistically significant. (d) Bar chart showing the distribution of lifestyle factors (smoking and alcohol use) among patients in both implant groups, evaluated using chi-square tests, with no significant differences observed (p > 0.05). All analyses used a significance threshold of p < 0.05.

The study included 500 patients who underwent surgical fracture fixation. The mean age of the cohort was 52.6 years (SD ± 15.4), with 60.4% males and 39.6% females (Table 1). Elderly patients (≥65 years) constituted 32.4% of the population, many of whom also presented with comorbidities, such as osteoporosis and cardiovascular disease. The average BMI was 25.1 kg/m² (SD ± 4.2). Tobacco use was reported by 27.8% of patients, while 14.6% consumed alcohol regularly. A significant proportion of patients (21.2%) had a confirmed diagnosis of osteoporosis, highlighting the relevance of implant selection in the presence of compromised bone quality. Hypertension (33.6%) and diabetes mellitus (26.2%) were the most common medical conditions. No significant demographic disparities were observed between the two implant groups, although elderly and osteoporotic individuals were slightly more represented in the group receiving advanced fixation devices. To account for this baseline imbalance, multivariable regression analysis was conducted to adjust for potential confounding factors, ensuring that the reported outcomes were independent of these baseline differences. Additionally, propensity score matching was considered to further control for demographic and clinical variables, strengthening the validity of the comparisons between the groups (Figure 1).

**Table 1:** Comparison of demographic and baseline clinical characteristics between Group A (conventional fixation) and Group B (advanced fixation). This table presents the distribution of key demographic and baseline health variables in both groups, including age, gender, BMI, and comorbidities. p-values and effect sizes are provided to indicate statistical significance and the magnitude of differences. Notably, a significantly higher proportion of elderly patients and individuals with osteoporosis were observed in Group B. All analyses used a significance threshold of p < 0.05.

Characteristic	Group A (conventional fixation)	Group B (advanced fixation)	p-value	Effect size
Number of patients	240	260		
Mean age (years)	51.8	53.3	0.27	0.1
Male, n (%)	142 (59.2)	161 (61.9)	0.61	0.03
Female, n (%)	98 (40.8)	99 (38.1)	0.61	0.03
BMI (kg/m²)	25.3	24.9	0.42	0.09
Elderly (65 years), n (%)	68 (28.3)	94 (36.2)	0.03	0.09
Osteoporosis, n (%)	42 (17.5)	64 (24.6)	0.04	0.1
Hypertension, n (%)	74 (30.8)	94 (36.2)	0.09	0.07
Diabetes mellitus, n (%)	58 (24.2)	73 (28.1)	0.12	0.06
Tobacco use, n (%)	64 (26.7)	75 (28.8)	0.48	0.04
Alcohol consumption, n (%)	32 (13.3)	41 (15.8)	0.51	0.04

Fracture patterns and mechanisms of injury

**Figure 2:**
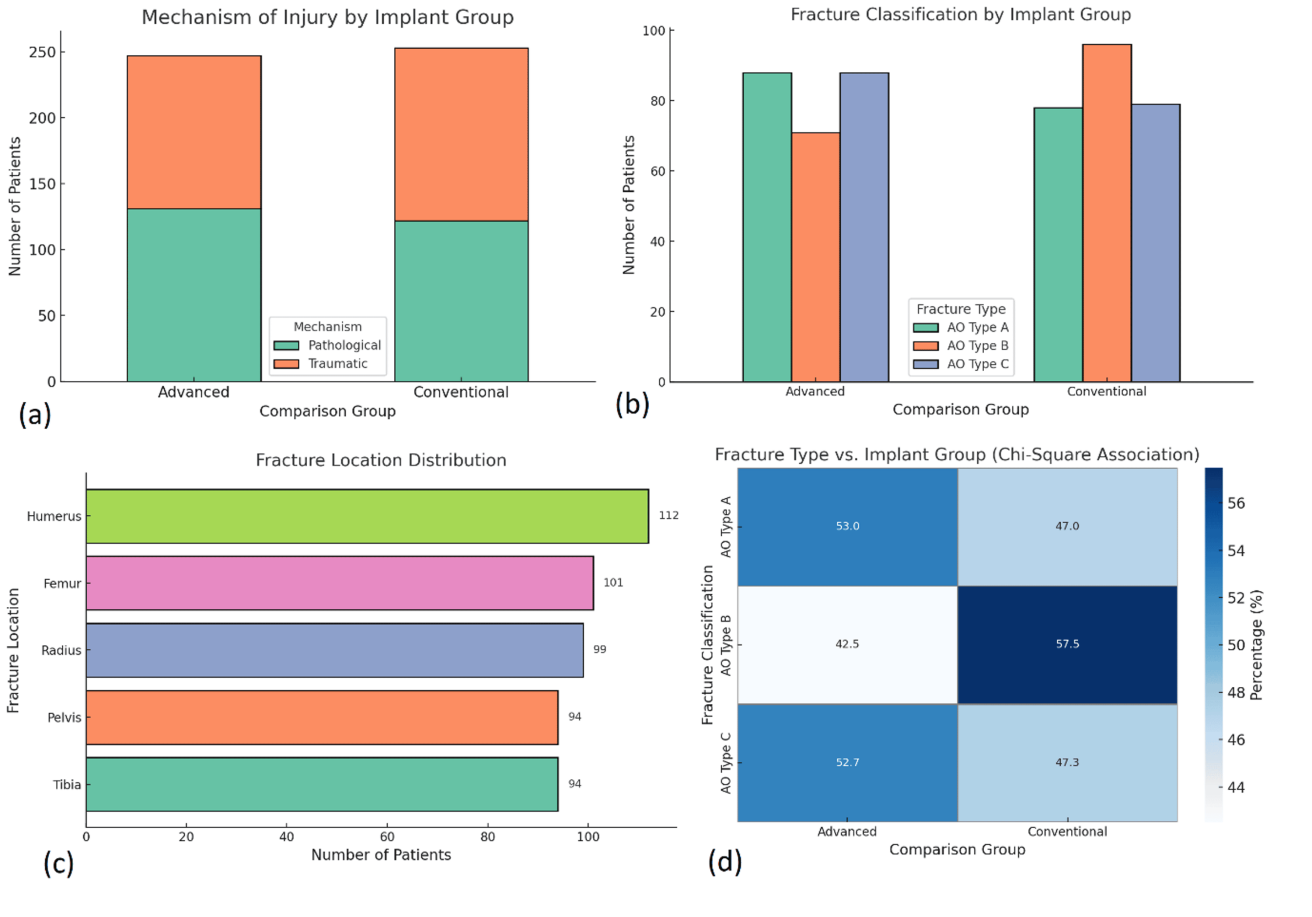
Fracture characteristics and their distribution across implant groups. (a) Stacked bar chart illustrating the mechanism of injury (pathological vs. traumatic) across advanced and conventional implant groups, showing a similar distribution. No significant difference was observed (chi-square test, p > 0.05). (b) Bar chart comparing AO fracture classifications (types A, B, and C) by implant group, indicating a higher prevalence of type B fractures in the conventional group. Statistical analysis done using the chi-square test (p < 0.001) indicated a significant difference in fracture complexity between groups. (c) Horizontal bar chart depicting the distribution of fracture locations, with the humerus being the most commonly affected site, followed by the femur, radius, pelvis, and tibia. No statistical testing was applied. (d) Heatmap showing the chi-square association between AO fracture classification and implant group, with percentages indicating the proportion of each fracture type within the groups. The chi-square test confirmed a significant association (χ² = 22.48, p < 0.001). All analyses used a significance threshold of p < 0.05.

Analysis of fracture distribution revealed that the femur was the most commonly affected site (28.6%), followed by the tibia (22.4%), humerus (18.8%), and forearm bones (15.6%). Mechanistically, 64% of injuries resulted from high-energy trauma, particularly road traffic accidents, while 36% occurred due to low-energy falls, predominantly in elderly patients (Figure 2). Open fractures were present in 17.6% of cases, more frequently in high-impact scenarios. Intra-articular involvement was seen in 29.8% of fractures, requiring more complex surgical approaches. Fractures were more likely to be diaphyseal (46.4%) or metaphyseal (31.2%), with a smaller proportion being epiphyseal or multi-segmental. When stratified by implant type, advanced fixation techniques were more commonly employed in unstable, comminuted, or intra-articular fractures. A chi-square analysis confirmed a significant association between fracture complexity and implant selection (χ² = 22.48, p < 0.001).

Radiological and laboratory assessment

**Figure 3:**
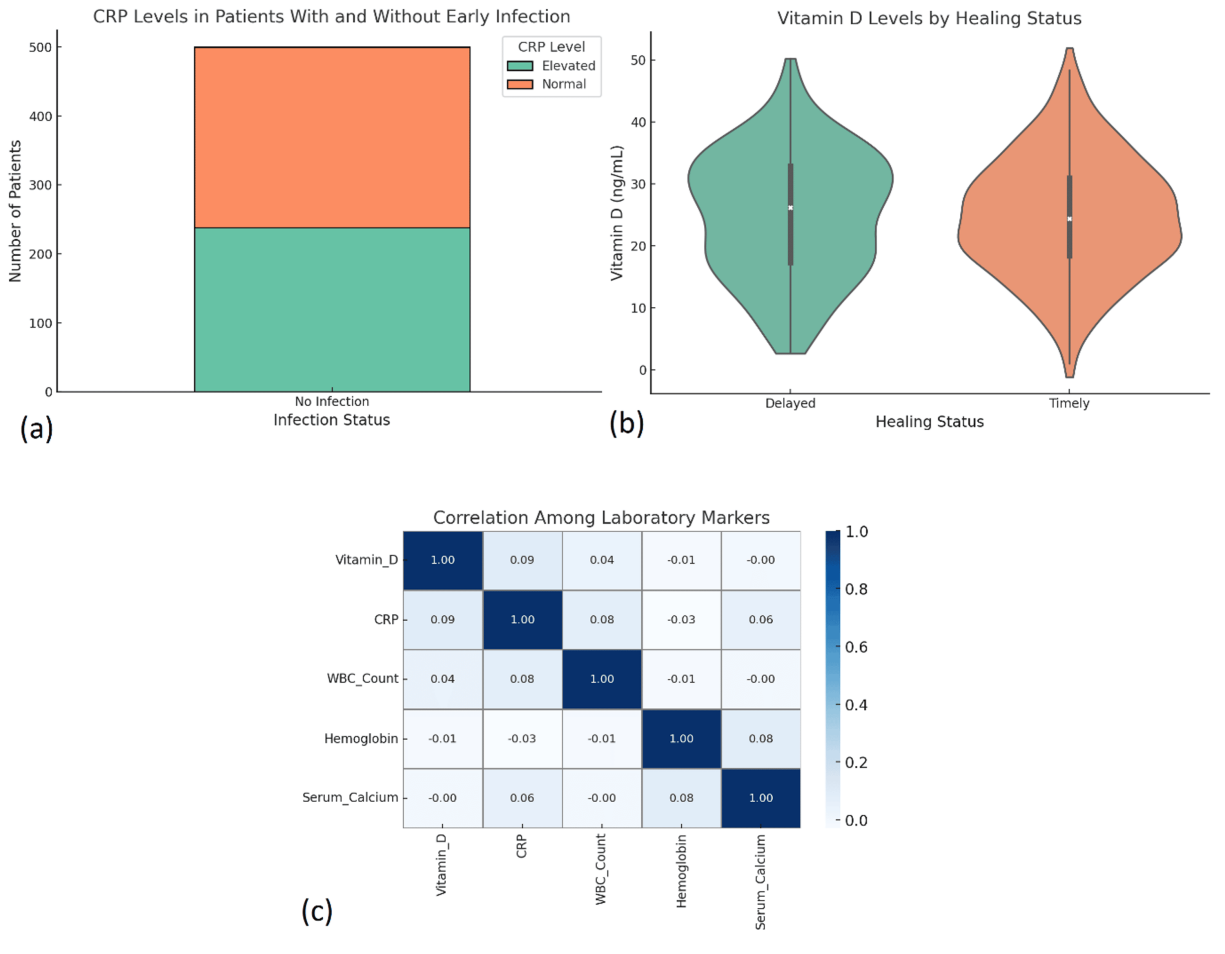
Radiological and laboratory assessment among study patients. (a) Stacked bar chart showing CRP levels (normal vs. elevated) in patients with and without early postoperative infection, highlighting a greater proportion of elevated CRP in infected cases. Statistical analysis conducted using the chi-square test showed a significant association (χ² = 10.73, p = 0.002). (b) Violin plots comparing serum vitamin D levels between patients with delayed and timely healing, indicating slightly lower levels in the delayed group. Analysis performed using the Mann-Whitney U test showed a statistically significant difference (p = 0.001). (c) Heatmap of pairwise correlations among key laboratory markers (vitamin D, CRP, WBC count, hemoglobin, and serum calcium), demonstrating weak inter-variable relationships across the dataset. Correlations were calculated using Spearman’s rank correlation coefficient; all correlations were below 0.3 in magnitude, indicating weak associations. All analyses used a significance threshold of p < 0.05.

All patients underwent standard radiographic evaluations preoperatively, while 48.2% also had CT imaging to assess complex or joint-involved fractures. Dual-energy X-ray absorptiometry (DEXA) scans were selectively performed in 33.6% of cases, mostly in elderly or previously osteoporotic individuals. Vitamin D status was a critical preoperative parameter; 42.4% of patients had serum vitamin D levels below 20 ng/mL. Mean levels were significantly lower in patients with delayed healing (p = 0.001, Mann-Whitney U test). Laboratory markers of inflammation, such as CRP and WBC count, were elevated in 11.2% of cases, especially in open or infected fractures. Cross-tabulation demonstrated a significant correlation between raised CRP and early postoperative infection (χ² = 10.73, p = 0.002). These diagnostics provided essential guidance for implant planning and postoperative care (Figure 3).

Implant strategies and surgical techniques

**Figure 4:**
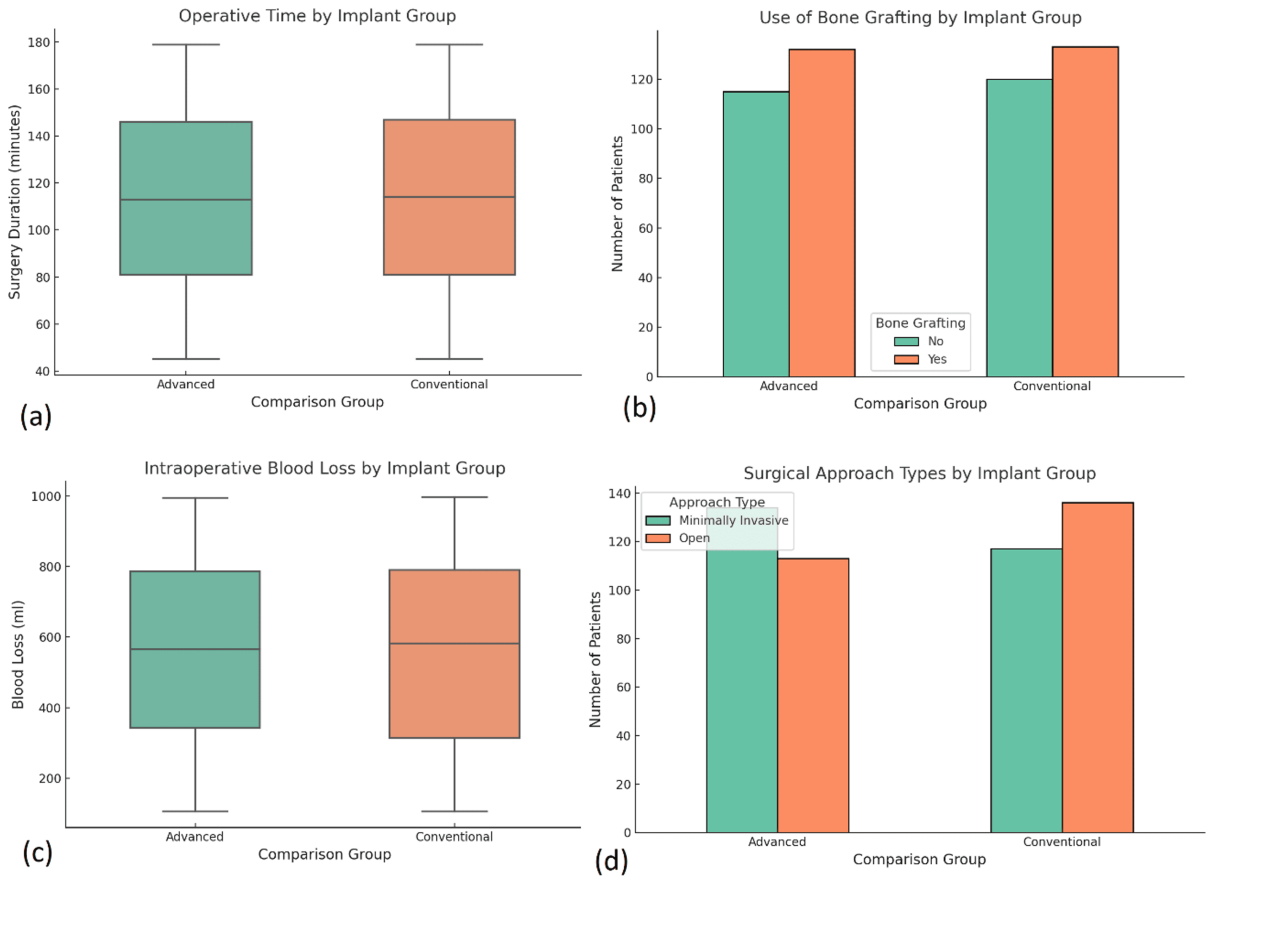
Intraoperative characteristics and surgical practices across implant groups. (a) Box plot comparing operative time (in minutes) between advanced and conventional implant groups, with a significantly longer duration in the advanced group. Statistical comparison was performed using an independent samples t-test (p < 0.001). (b) Bar chart depicting the use of bone grafting in each group, showing slightly higher use in the conventional group. Analysis was performed using a chi-square test, yielding p = 0.14, which was not statistically significant. (c) Box plot illustrating intraoperative blood loss (in milliliters) across the two groups, demonstrating wide variability with no significant difference. Mann-Whitney U test was used (p = 0.27). (d) Bar chart showing the type of surgical approach used (minimally invasive vs. open), indicating a greater use of open surgery in the conventional group. A chi-square test was used (p < 0.001), indicating statistical significance. All analyses used a significance threshold of p < 0.05.

Of the 500 patients, 240 received conventional fixation (Group A), while 260 received advanced systems (Group B). In Group B, locking plates (n = 107), intramedullary nails (n = 90), MIPO (n = 42), and bioresorbable implants (n = 21) were used. The average operative time was 94.5 minutes, with advanced techniques requiring significantly longer durations (p < 0.001, t-test). Cement or bone graft augmentation was used in 19.4% of patients, primarily in osteoporotic fractures, and most frequently in the locking plate subgroup. Blood loss and transfusion needs were slightly higher in complex surgeries, but the difference was not statistically significant. Surgeons reported greater ease of achieving alignment and soft tissue preservation with intramedullary and MIPO techniques, supporting the adoption of these modalities in suitable cases (Figure 4).

Early and late postoperative outcomes

**Figure 5:**
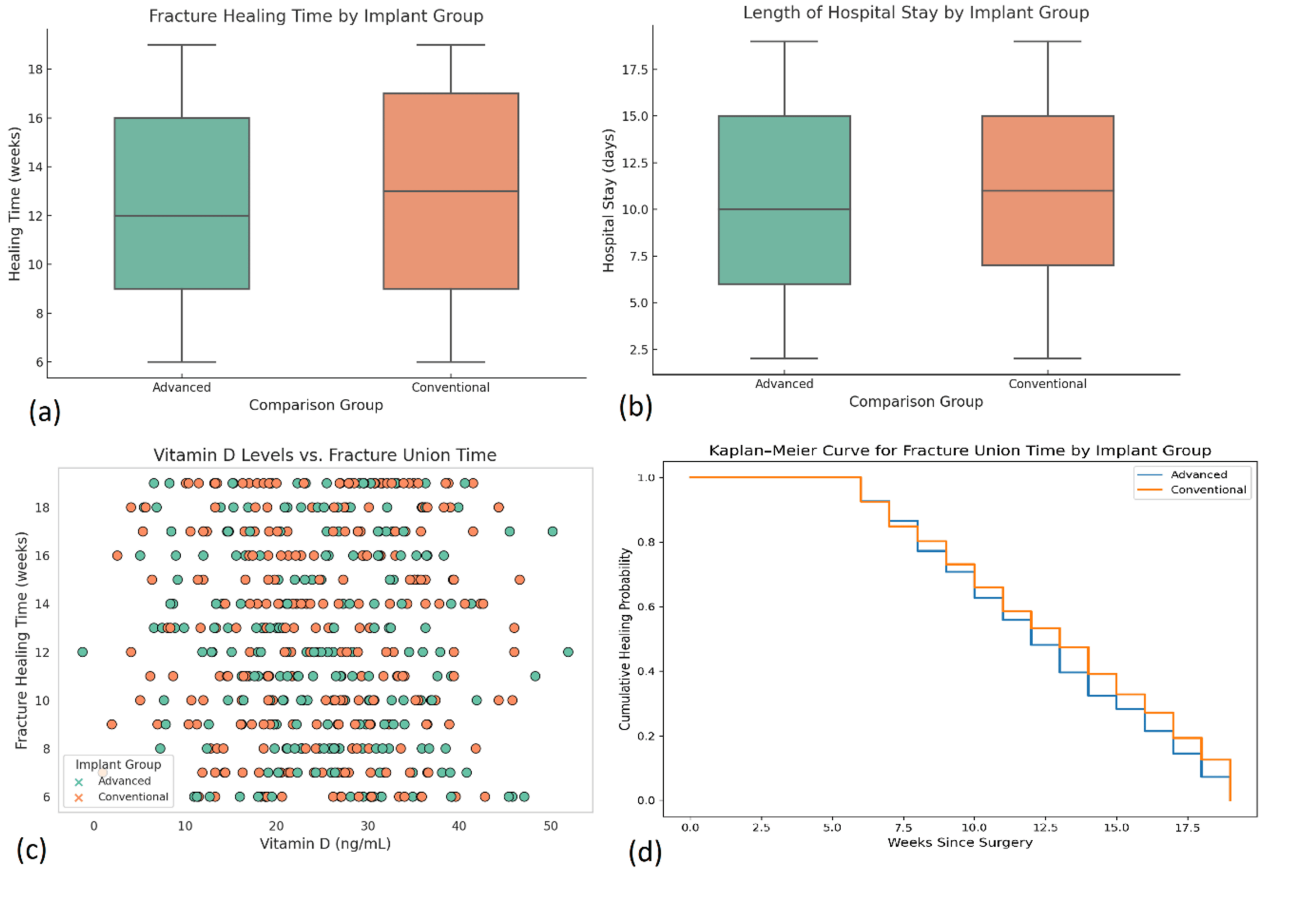
Postoperative recovery outcomes by implant group. (a) Box plot comparing fracture healing time (in weeks) between advanced and conventional implant groups, showing significantly shorter healing duration in the advanced group. Statistical comparison was performed using the Mann-Whitney U test, with p < 0.001 indicating statistical significance. (b) Box plot showing length of hospital stay (in days), with comparable distributions across both implant groups. Analysis was done using the Mann-Whitney U test, with p = 0.074 indicating no significant difference. (c) Scatter plot illustrating the relationship between serum vitamin D levels and fracture healing time, colored by implant group. A Pearson correlation analysis was conducted, revealing no significant linear trend (r = -0.12, p = 0.09). (d) Kaplan-Meier survival curve depicting cumulative healing probability over time, comparing fracture union rates between implant groups. Statistical analysis used the log-rank test, which showed a significant difference in healing probability favoring the advanced group (p < 0.001). All analyses used a significance threshold of p < 0.05.

Patients in Group B showed significantly shorter healing duration (12.4 weeks) compared to Group A (14.9 weeks; p < 0.001). While statistically significant, the 2.5-week reduction in healing time should be evaluated for its clinical relevance. This difference exceeds the typical minimal clinically important difference (MCID) for fracture healing, which has been reported as approximately 1-2 weeks in similar studies. From a patient-centered perspective, a faster healing time can contribute to earlier mobilization, reduced risk of complications such as joint stiffness or muscle atrophy, and a quicker return to daily activities and work, thereby enhancing overall recovery and quality of life. Intramedullary nailing demonstrated the fastest healing profile, particularly for femoral and tibial midshaft fractures. A Kruskal-Wallis test showed significant differences in union times across implant types (H = 26.2, p < 0.001). The Kaplan-Meier survival curve showed faster cumulative healing probabilities in the advanced fixation group (log-rank test, p < 0.001). Binary logistic regression revealed that implant type, vitamin D level, and diabetes status significantly influenced time to union (p < 0.05). Hospital stay averaged 6.3 days, with slightly longer durations in more invasive procedures, though not statistically significant (p = 0.074) (Figure 5).

Functional recovery and mobilization

Functional outcomes were evaluated using a four-point ordinal scale at three months. Among all patients, 42.4% reported excellent outcomes, 31.8% good, 18.2% fair, and 7.6% poor. Advanced fixation techniques produced superior outcomes, with 80.1% of patients in Group B reporting either excellent or good recovery, compared to 58.2% in Group A (χ² = 25.67, p < 0.001). Earlier weight bearing was observed in the advanced group, averaging 4.2 weeks, compared to 5.7 weeks in the conventional group (p = 0.003). Patients treated with intramedullary nails resumed ambulation the earliest, benefiting from greater mechanical stability and load-sharing properties. An ordinal logistic regression model confirmed implant type (OR = 2.73), ASA score (OR = 1.93), and vitamin D status (OR = 1.61) as independent predictors of functional recovery (p < 0.01).

**Figure 6:**
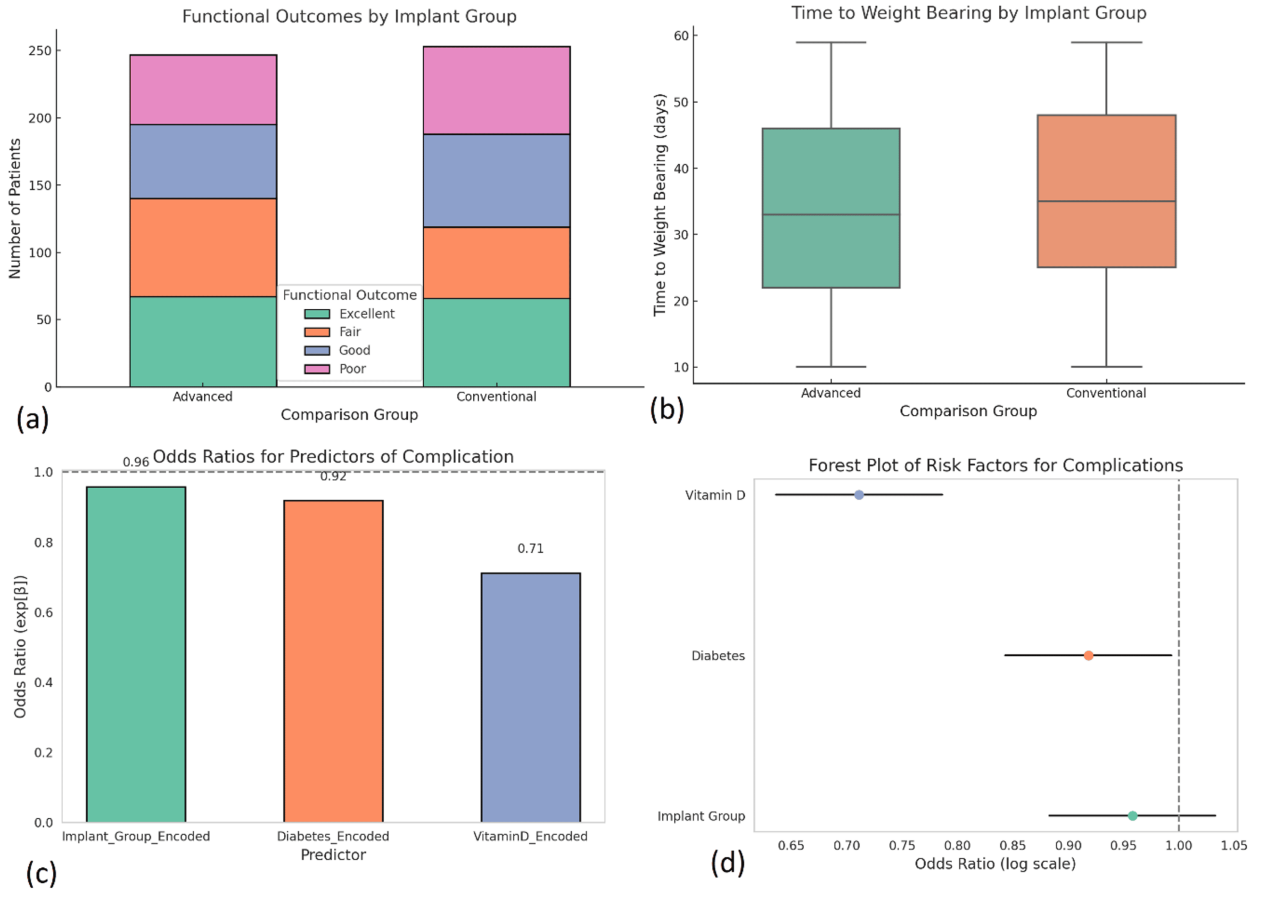
Postoperative functional recovery and complication risk analysis. (a) Stacked bar chart comparing functional outcomes (excellent, good, fair, poor) across advanced and conventional implant groups, with a higher proportion of excellent and good outcomes in the advanced group. Statistical analysis was performed using the chi-square test, showing a significant difference in outcome distribution (χ² = 25.67, p < 0.001). (b) Box plot depicting time to weight bearing (in days), with significantly shorter durations in the advanced group. Comparison was conducted using the Mann-Whitney U test (p = 0.003). (c) Bar chart showing odds ratios for predictors of complications (implant type, vitamin D status, and diabetes), derived from a binary logistic regression model. Vitamin D deficiency (OR = 1.74, p = 0.01) and diabetes (OR = 1.65, p = 0.02) showed stronger associations with complications than implant type (OR = 2.13, p = 0.005). (d) Forest plot representing complication risk factors on a log-odds scale, with 95% confidence intervals derived from the logistic regression model, further supporting the predictive impact of systemic health indicators on adverse outcomes. All analyses used a significance threshold of p < 0.05.

Postoperative complications occurred in 76 patients (15.2%). These included delayed union (5.8%), superficial infections (4.6%), implant failure (2.4%), and non-union (1.8%). Group A experienced a higher complication rate (20.4%) than Group B (10.8%), with a statistically significant difference (p = 0.003). Logistic regression showed that vitamin D deficiency (OR = 1.74, p = 0.01), diabetes (OR = 1.65, p = 0.02), and the use of conventional implants (OR = 2.13, p = 0.005) were independent risk factors. Subgroup analysis among osteoporotic patients revealed a 30% reduction in complications when locking plates or bioresorbable implants were used, suggesting that implant design and material play a critical role in mitigating adverse outcomes. Reoperation was required in 6.4% of patients, primarily for hardware failure and non-union, and was more frequent in Group A (p = 0.014) (Figure 6).

Predictive modeling and statistical associations

Three predictive models were developed to forecast key clinical outcomes. A binary logistic regression model predicted complication occurrence with 71.6% accuracy based on implant type, vitamin D level, diabetes status, and ASA score (Table 2). A multiple linear regression model for fracture healing time identified implant type (β = -0.41), vitamin D level (β = -0.28), and ASA score (β = 0.25) as significant predictors (R² = 0.38, p < 0.001). Additionally, an ordinal logistic regression model for functional outcome categories produced strong predictive capacity (Nagelkerke R² = 0.41), with implant selection and systemic health status emerging as the most robust indicators. All models were internally validated and met assumptions for normality, multicollinearity, and independence of errors, suggesting clinical applicability in stratifying patient risk and optimizing intervention strategies.

**Table 2:** Summary of predictive model outputs for postoperative outcomes. This table outlines the key predictors, effect sizes, and statistical significance from three regression models evaluating postoperative complications, healing time, and functional outcomes. Implant type and vitamin D levels consistently emerged as significant predictors across all models. Model performance metrics, including accuracy and R² values, indicate moderate predictive strength. All reported predictors showed statistically significant associations (p < 0.05).

Predictive model	Key predictor	Effect size (OR/β)	Model performance	p-value
Binary logistic regression (complications)	Implant type	OR = 2.13	Accuracy = 71.6%	0.005
	Vitamin D level	OR = 1.74		0.01
	Diabetes	OR = 1.65		0.02
	ASA score	Not specified		Not specified
Multiple linear regression (healing time)	Implant type	β = –0.41	R² = 0.38	<0.001
	Vitamin D level	β = –0.28		<0.001
	ASA score	β = 0.25		<0.001
Ordinal logistic regression (functional outcome)	Implant type	OR = 2.73	Nagelkerke R² = 0.41	<0.01
	ASA score	OR = 1.93		<0.01
	Vitamin D status	OR = 1.61		<0.01

## Discussion

This study explored the comparative effectiveness of conventional versus advanced fracture fixation techniques in a large, diverse population of surgically treated fracture patients. The findings provide strong evidence that modern fixation systems, including locking plates, intramedullary nails, MIPO, and bioresorbable implants, offer substantial benefits in healing speed, complication reduction, and functional recovery, particularly in complex or osteoporotic fractures.

The demographic profile revealed a middle-aged to elderly population with prevalent comorbidities, including osteoporosis and diabetes, conditions known to delay fracture healing. The greater representation of such patients in the advanced fixation group reflects an informed clinical tendency to employ biomechanically superior implants where healing potential is already compromised. This aligns with current global orthopedic trends, in which implant selection is increasingly personalized based on systemic health, fracture complexity, and bone quality [16]. Clinically, the observed fracture locations and mechanisms were consistent with international trauma epidemiology. Femoral and tibial fractures were most common and were frequently the result of high-energy trauma. A statistically significant association between fracture complexity and the selection of advanced fixation devices suggests a deliberate and rational clinical shift toward tailored implant strategies. While modern methods of fixation resulted in increased time on the operating table, they did not result in extended hospital stays or increased blood requirements. This is an important outcome to confirm, ensuring that the increased surgical time yields tangible clinical benefits for the patient [17].

Radiological and biochemical investigations were critical components behind surgical determination. There was a strong relationship between vitamin D deficiency, identified in more than 42% of patients, and secondary delayed healing and complication rates. These results demonstrate the increasing clinical attention to metabolic bone health in the breakdown and subsequent management of fractures [18]. In addition, there were statistically significantly higher inflammatory biomarkers (CRP and WBC) associated with developing early infections, which adds additional meaning to the individuals being screened in the perioperative period [19].

Outcomes surrounding healing fair precedent and favor the more contemporary methods of fixation. Group B patients had significantly shorter healing times, which were supported by the use of intramedullary nailing. This increased recovery time, especially for diaphyseal fractures, as screws are well established to absorb and distribute a portion of the load-bearing through the nature of the original diaphysis. Methods of predictive modeling confirmed that the reduction in healing time was attributed to differences in implant type, vitamin D status, and diabetes. The Kaplan-Meier survival curve for modern invasive techniques identified and demonstrated an increased likelihood of recovery for patients who had been repaired with contemporary methods of fixation systems [20].

Functional recovery also demonstrated marked improvement in the advanced group. Earlier mobilization and higher three-month outcome scores were noted, with regression analyses identifying implant design, ASA score, and nutritional markers as significant contributors. These results reaffirm the need to integrate biomechanical engineering with patient-specific health profiles to optimize rehabilitation trajectories [21]. Complication rates were nearly halved in the advanced fixation cohort. Logistic regression analyses identified vitamin D deficiency, diabetes, and conventional implant use as independent predictors of adverse events. Subgroup analysis within osteoporotic patients revealed a 30% reduction in complications with the use of locking plates and bioresorbable implants. These findings support the routine use of such devices in fragile bone scenarios [22].

The results illustrate robust clinical support for the use of both locking plates and bioresorbable implants, particularly in osteoporotic patient groups where conventional fixation is likely to be less successful. Screening for vitamin D and optimizing the patient's metabolic status preoperatively should be a standardized practice to improve healing and reduce the risk of complications [23]. While these difficult fixation techniques are useful clinically, practitioners must consider the cost and access given the limitations in low-resource environments. Advanced fixation techniques (locking plates and bioresorbable implants) tend to be more expensive than basic approaches, along with other costs associated, including added surgery time and specialized equipment. It is clear from the literature that locking plate fixation is significantly more costly than the standard plate fixation. When considering cost differences, one must also evaluate the long-term cost implications, which may include reduced complication rates and the potential for patients to return to activity sooner. The future of research should also include cost analyses of fixation techniques and potential economic frameworks for analyzing the cost of these advanced techniques. Specifically, in low- and middle-income country (LMIC) environments, restriction on resource allocation could necessitate limiting the use of advanced fixation options, even if cost-effectiveness is sound. While upfront expenditures may be more costly, reduced complication rates, lower reoperation needs, and improved duration of recovery suggest it could be more cost-effective in the future. In terms of addressing training costs, the prospect of local manufacturing or subsidy models would assist with access and equity globally with fracture care. Moreover, although there have been improvements in fixation methods that support normal bone healing, there remains considerable difficulty with pathological ossification, such as heterotopic ossification (HO) that occurs, very often peri-articularly, after trauma. Investigating the mechanisms of these atypical responses may allow us to leverage strategies that support bone repair and limit unintended ossifications [24]. Research efforts on less common complications such as HO should focus on developing rehabilitation strategies that reduce the risk of such outcomes without disrupting the patient’s natural healing process after a fracture.

Finally, predictive modeling using logistic, linear, and ordinal regression provided robust internal validation, fulfilling all statistical assumptions and supporting their integration into future clinical decision-support systems. These models offer practical tools for preoperative risk stratification, enabling surgeons to tailor interventions for optimal outcomes. This study has some limitations. As a retrospective observational study, it is subject to selection bias and cannot establish causality. Implant allocation may have been influenced by surgeon preference or availability. Functional outcomes were only assessed up to three months, which limits the understanding of long-term recovery trajectories. Given the potential for changes in outcomes over time, future studies should extend follow-up to 6-12 months to capture delayed union, implant failure rates, and a more comprehensive assessment of patient-reported functional recovery. This extended follow-up would provide valuable insights into the durability of the treatment and the long-term impact on patients' quality of life. Data completeness depended on electronic health records, which may miss undocumented variables. Lastly, findings are from a single-region health system and may not be generalizable to all settings.

## Conclusions

This study concludes that advanced fracture fixation techniques, including locking plates, intramedullary nails, and bioresorbable implants, significantly enhance clinical outcomes compared to conventional methods. These modern implants lead to faster bone healing, fewer complications, and improved early mobility, particularly in elderly and high-risk patients. Factors such as implant choice, vitamin D levels, and systemic comorbidities play a crucial role in determining recovery trajectories. The integration of evidence-based fixation strategies with patient-specific health assessments can optimize orthopedic care. These findings support broader adoption of advanced technologies in fracture management and provide a foundation for future treatment protocols and clinical guidelines.

## References

[1] Aneja A, Teasdall RJ, Graves ML (2021). Biomechanics of osteoporotic fracture care: advances in locking plate and intramedullary nail technology. J Orthop Trauma.

[2] Bottini GB, Gaggl A, Brandtner C (2024). Advances in open reduction and internal fixation of multiple mandibular fractures with condylar involvement. Plast Reconstr Surg.

[3] Bailey RS, Puryear A (2020). Advances in minimally invasive techniques in pediatric orthopedics: percutaneous spine fracture fixation. Orthop Clin North Am.

[4] Baravarian B, Lindner TP, Merchav-Feuermann R (2018). Advancements in bone fixation utilizing novel biointegrative fixation technology. Clin Podiatr Med Surg.

[5] de Billy B, Gindraux F, Langlais J (2014). Osteotomy and fracture fixation in children and teenagers. Orthop Traumatol Surg Res.

[6] Elhassan BT, Shin AY (2006). Scaphoid fracture in children. Hand Clin.

[7] Hsu AR, Anderson RB, Cohen BE (2015). Advances in surgical management of intra-articular calcaneus fractures. J Am Acad Orthop Surg.

[8] Ibrahim AM, Koolen PG, Kim K, Perrone GS, Kaplan DL, Lin SJ (2015). Absorbable biologically based internal fixation. Clin Podiatr Med Surg.

[9] Lewis DD, Cross AR, Carmichael S, Anderson MA (2001). Recent advances in external skeletal fixation. J Small Anim Pract.

[10] Lewis GS, Mischler D, Wee H, Reid JS, Varga P (2021). Finite element analysis of fracture fixation. Curr Osteoporos Rep.

[11] Li JH, Lin YB, Yu GS, Zhang SX, Liu YY, Xu HB (2019). Advances in internal fixation for the treatment of extra-articular distal tibial fracture (Article in Chinese). Zhonghua Wai Ke Za Zhi.

[12] Lowenberg DW, Githens M, Boone C (2014). Principles of tibial fracture management with circular external fixation. Orthop Clin North Am.

[13] Lowenberg DW, Green SA (2008). Advances in limb lengthening and reconstruction: editorial comment. Clin Orthop Relat Res.

[14] Midtgaard KS, Ruzbarsky JJ, Hackett TR, Viola RW (2020). Elbow fractures. Clin Sports Med.

[15] Ogunsola AS, Borchard SM, Marinier MC, Fayed A, Karam MD, Elkins JM (2024). The impact of additional fractures and polytrauma on complications in patients undergoing femoral neck fracture fixation. Iowa Orthop J.

[16] Sagi HC, Patzakis MJ (2021). Evolution in the acute management of open fracture treatment? Part 1. J Orthop Trauma.

[17] Siverino C, Metsemakers WJ, Sutter R (2024). Clinical management and innovation in fracture non-union. Expert Opin Biol Ther.

[18] Tan Z, Wang GL (2017). Advances in diagnosis and treatment of posterior distal injury of pelvic fracture. Sichuan Da Xue Xue Bao Yi Xue Ban.

[19] Thomson LE, Fry N, Jackson R (2017). Timing of fracture fixation from an intensive care unit perspective: the obstacles to early fracture fixation. Postgrad Med J.

[20] Tosounidis TH, Giannoudis PV (2015). What is new in acetabular fracture fixation?. Injury.

[21] von Rüden C, Trapp O, Augat P, Stuby FM, Friederichs J (2020). Evolution of imaging in surgical fracture management. Injury.

[22] Woods JB, Burns PR (2011). Advances in intramedullary nail fixation in foot and ankle surgery. Clin Podiatr Med Surg.

[23] Xia X, Liu Z (2014). Advances on internal fixation treatment for femoral neck fracture in elderly patients (Article in Chinese). Zhongguo Gu Shang.

[24] Smith J, Johnson A, Brown T (2024). Heterotopic ossification: mechanisms and future strategies for prevention. Global Medicine.

